# Endothelial Senescence and Chronic Fatigue Syndrome, a COVID-19 Based Hypothesis

**DOI:** 10.3389/fncel.2021.673217

**Published:** 2021-06-25

**Authors:** Adonis Sfera, Carolina Osorio, Carlos M. Zapata Martín del Campo, Shaniah Pereida, Steve Maurer, Jose Campo Maldonado, Zisis Kozlakidis

**Affiliations:** ^1^Patton State Hospital, San Bernardino, CA, United States; ^2^Loma Linda University, Loma Linda, CA, United States; ^3^Psychiatry Service, Outpatient Consultation Department, National Institute of Cardiology Ignacio Chavez, Mexico, Mexico; ^4^Department of Internal Medicine, The University of Texas Rio Grande Valley, Edinburg, TX, United States; ^5^International Agency for Research on Cancer (IARC), Lyon, France

**Keywords:** endothelial cells, cellular senescence, gut microbial community, endotoxin tolerance, microbial translocation

## Abstract

Myalgic encephalomyelitis/chronic fatigue syndrome is a serious illness of unknown etiology, characterized by debilitating exhaustion, memory impairment, pain and sleep abnormalities. Viral infections are believed to initiate the pathogenesis of this syndrome although the definite proof remains elusive. With the unfolding of COVID-19 pandemic, the interest in this condition has resurfaced as excessive tiredness, a major complaint of patients infected with the SARS-CoV-2 virus, often lingers for a long time, resulting in disability, and poor life quality. In a previous article, we hypothesized that COVID-19-upregulated angiotensin II triggered premature endothelial cell senescence, disrupting the intestinal and blood brain barriers. Here, we hypothesize further that post-viral sequelae, including myalgic encephalomyelitis/chronic fatigue syndrome, are promoted by the gut microbes or toxin translocation from the gastrointestinal tract into other tissues, including the brain. This model is supported by the SARS-CoV-2 interaction with host proteins and bacterial lipopolysaccharide. Conversely, targeting microbial translocation and cellular senescence may ameliorate the symptoms of this disabling illness.

## Introduction

Excessive accumulation of senescent cells in body tissues has been associated with organismal aging and fatigue as observed in older individuals and patients treated with anticancer agents (Sanoff et al., 2014; Rajeevan et al., 2018; Xu et al., 2018). On the other hand, preclinical studies have reported that the selective elimination of senescent cells could alleviate not only some chemotherapy adverse effects but also various age-related symptoms, including muscle weakness, fatigue and frailty, suggesting a potential treatment modality for myalgic encephalomyelitis/chronic fatigue syndrome (ME/CFS) (Demaria et al., 2017; Short et al., 2019; Kaur et al., 2020; NCT03675724).

Long lasting myalgia and exhaustion were reported in over 40% of COVID-19 patients, indicating that the SARS-CoV-2 virus may directly invade skeletal muscles, triggering myositis (Chen G. et al., 2020; Huang et al., 2020; Kucuk et al., 2020). Indeed, as muscle cells express abundant angiotensin converting enzyme-2 (ACE-2), the virus likely exploits the myocytes, engendering disabling symptoms such as fatigue and weakness (Ferrandi et al., 2020; Jin and Tong, 2020; Mao et al., 2020). For example, preclinical studies have demonstrated that SARS-CoV-2-upregulated angiotensin II (ANG II) disrupts the muscle cell autophagy, impairing both the metabolism and contractility (Neel et al., 2013; Silva et al., 2019). However, aside from directly accessing the myocytes, this virus can induce myopathy, muscle weakness and atrophy indirectly by upregulating proinflammatory cytokines, such as interleukin 1 beta (IL-1β) and 6 (IL-6), C-reactive protein (CRP), and tumor necrosis factor (TNF) (VanderVeen et al., 2019; Guidon and Amato, 2020; Jin and Tong, 2020). When persistent, exertional and unrelieved by rest, myopathy may gradually morph into ME/CFS, a severe illness, affecting up to 2.5 million Americans (Blomberg et al., 2018; Friedman, 2019).

The National Academy of Medicine (former Institute of Medicine) 2015 ME/CFS diagnostic criteria include 6 months or longer of post-exertional malaise and unrefreshing sleep along with either cognitive impairment or orthostatic intolerance (Cortes Rivera et al., 2019). Tiredness and exhaustion are non-specific symptoms that may be experienced as central or “brain fog,” muscular and post-infectious, emphasizing the multifactorial nature of this condition (Greenberg, 2002; Yamashita, 2020). Indeed, the ME/CFS etiology includes genetic pre-disposition, inflammation, metabolic dysfunction, gastrointestinal pathology, autoimmunity, and viral infections (Jason et al., 1999; Ortega-Hernandez and Shoenfeld, 2009; Scherbakov et al., 2020).

**Figure 1:**
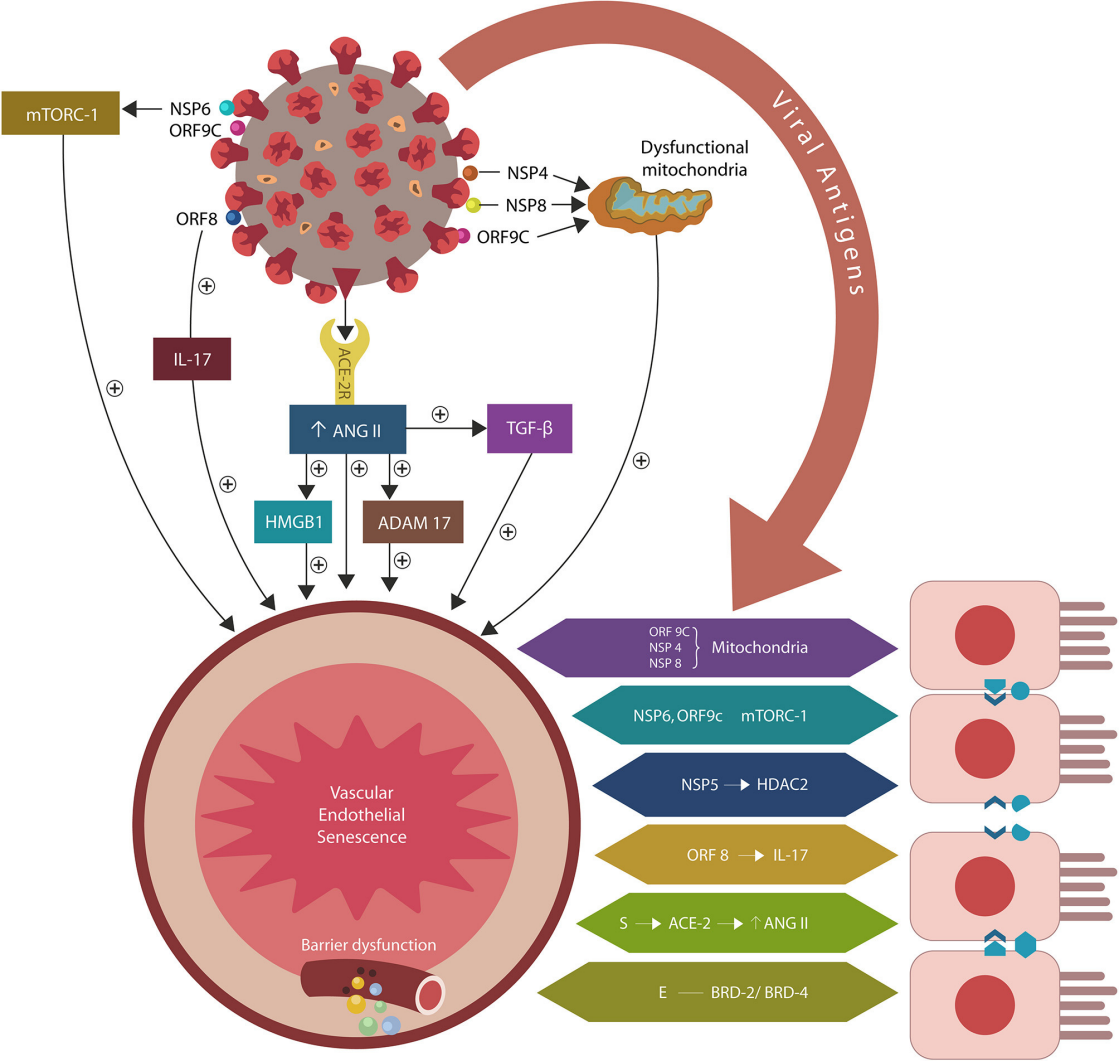
SARS-CoV-2/host protein-protein interactions. Viral crosstalk with several host proteins disrupts both endothelial cells (ECs) and intestinal epithelial cells (IECs), damaging the gut barrier and facilitating microbial and/or lipopolysaccharide (LPS) translocation into host tissues, including the skeletal muscle and the brain. Endothelial senescence also contributes to the disruption of blood-brain barrier (BBB), allowing microbial and/LPS access into the CNS. It is noteworthy that the cross talk between viral E antigen and host bromodomains (BRDs) 2 and 4 triggers macrophage senescence, impairing the elimination (efferocytosis) of aging cells. Viral S antigen attachment to ACE-2 receptor (ACE-2R) is followed by ANG II upregulation. This augments TGF-β, HMGB1, ADAM17, and ICAM-1 (not shown), inflicting endothelial and intestinal cells dysfunction with barrier disruption.

During the COVID-19 pandemic, interest in ME/CFS has resurfaced as disabling fatigue, experienced by many patients, often lingers long after recovery, lowering the life quality (Wilson, 2020). Indeed, novel studies have reported ME/CFS in the aftermath of influenza and coronavirus infections, connecting viruses to the pathogenesis of severe exhaustion (Moldofsky and Patcai, 2011; Mohabbat et al., 2020; Poole-Wright et al., 2020). Along these lines, COVID-19 was demonstrated to alter several pathways previously associated with ME/CFS, suggesting that a better understanding of the virus/host interactome may elucidate the molecular underpinnings of this disease. Indeed, the viral crosstalk with several human proteins expressed by the intestinal epithelial cells (IECs) and endothelial cells (ECs) may alter the intestinal barrier, enabling microbial translocation from the gastro-intestinal (GI) tract into other tissues, including the brain (Maes and Leunis, 2008; Maes et al., 2014; Navaneetharaja et al., 2016; Proal and Marshall, 2018; Figure 1).

In an earlier paper, we proposed that premature EC senescence could increase the permeability of intestinal and blood-brain barrier (BBB), contributing to COVID-19 critical illness and its long-term sequelae (Sfera et al., 2020; Figure 1). Subsequent studies endorsed this model by demonstrating that aside from ACE-2, the SARS-CoV-2 virus can access host cells via dipeptidyl peptidase-4 (DPP4) and neuropilin-1 (NRP-1), receptors associated with ECs senescence (Kim et al., 2017; Issitt et al., 2019; Cantuti-Castelvetri et al., 2020; Chen Z. et al., 2020; Solerte et al., 2020). Further proof came from the reports linking the renin-angiotensin system (RAS) to accelerated aging via ANG II-induced telomere attrition demonstrated in both COVID-19 critical illness and Hutchinson-Gilford progeria (HGP) (Regenass et al., 1994; Herbert et al., 2008; Gerhard-Herman et al., 2012; Amraei and Rahimi, 2020; Aviv, 2020; Benetos et al., 2020; Bidault et al., 2020; Libby and Lüscher, 2020).

In the present hypothesis article, we surmise that COVID-19 sequelae, including the ME/CFS, may be caused by ANG II-inflicted fragmentation of biological barriers with subsequent microbial and/or lipopolysaccharide (LPS) translocation from the GI tract into various tissues, including the central nervous system (CNS). We focus primarily on the interaction between various SARS-CoV-2 antigens and host proteins expressed by ECs, IECs and immune cells that may increase barrier permeability, facilitating microbial translocation. If confirmed, this paradigm may open novel treatment opportunities in ME/CFS, including senotherapeutics as well as LPS and efferocytosis-targeting agents.

## The Overall Presentation of Biological Model of Action

According to this paradigm, the ME/CFS pathology is initiated by cellular senescence and barrier disruption promoted by the SARS-CoV-2-upregulated ANG II or by direct viral interaction with host proteins (Figure 1). When intestinal repair is delayed (due to specific host factors), the prolonged microbial translocation results in aberrant immune responses characteristic of both ME/CFS and COVID-19 critical illness (Loebel et al., 2016; Morris et al., 2019; Wang E. Y. et al., 2020; Wang L. et al., 2020).

The model presented here is supported by the SARS-CoV-2 interactome and the cross talk between microbial LPS and viral S protein that disrupt biological barriers (Gaab et al., 2005; Maes and Leunis, 2008; Giloteaux et al., 2016; Petruk et al., 2020). Indeed, recent studies in both humans and rodents linked LPS to the unexplained fatigue, myopathy, muscle wasting and memory impairment, likely implicating the endotoxin in the pathogenesis of ME/CFS (Langhans et al., 2014; Friedrich et al., 2015; Zhang et al., 2016; Batista et al., 2019; Lasselin et al., 2020). With the same token, LPS was recently connected to neurodegenerative disorders, indicating that this pathology may be initiated by the translocation of microbes and/or their molecules into the CNS (Pretorius et al., 2018; Zhan et al., 2018).

These aspects are presented below in more detail and in relation to the SARS-COV-2 virus, starting with the interactome and cellular senescence (both for ANG II-dependent and ANG II-independent molecular changes); ANG II and defective efferocytosis; and building the case toward the connection with gut biology. Lastly, the reviewed evidence is synthesized for potential therapeutic interventions, including senotherapeutic strategies.

## The Interactome and Cellular Senescence

The SARS-CoV-2 is an enveloped, positive-sense, single-stranded RNA virus that enters host cells via several receptors, including ACE-2, DPP-4 and NRP-1, expressed by various human tissues, including the gut, lung, muscle and ECs (Cantuti-Castelvetri et al., 2020; Fadini et al., 2020). The virus contains a genome of 30 kb that encodes for 29 proteins divided into structural, non-structural (NSP) and open-reading frame (ORF). These proteins were demonstrated to interact with as many as 332 human molecules, altering numerous pathways (Gordon et al., 2020).

The attachment of SARS-CoV-2 virus to ACE-2 receptors engenders ANG II dependent and independent molecular changes as reviewed below. The former include activation of ADAM17 (a disintegrin and metalloproteinase 17), ACE-2 downregulation and upregulation of transforming growth factor beta (TGF-β), high mobility group box 1 protein (HMBG1), toll-like receptor 4 (TLR4) and intercellular adhesion molecule 1 (ICAM-1). ANG II independent changes are comprised of direct interactions between viral antigens and host proteins that alter endothelia, skeletal muscle repair and immunological tolerance, especially the regulatory T cells (Tregs) and phagocytes [macrophages and natural killer cells (NKCs)] (Figure 1, Table 1).

**Table 1:** Angiotensin II independent changes: viral antigens interact directly with host proteins, altering several senescence pathways, including the metabolism (mitochondrial damage), telomeres and angiogenesis that disrupt biological barriers and immunity, likely contributing to ME/CFS.

**Interactome**	**Mitochondrial damage**	**Telomere attrition**	**Impaired angiogenesis**	**Senescence**	**Gut/Muscle pathology**	**Claudins/gut barrier**	**Myocytes/IECs**
Viral protein	ORF9C NSP4 NSP8	NSP5	E	S	N	ORF8	NSP6 ORF9C
Host protein	Mitochondrial proteins	HDAC2 GPX	BRD-2 BRD-4	NRP-1 DDP-4	STAT-1 STAT-2	IL-17	mTORC-1

### ANG II Dependent Molecular Changes

The SARS-CoV-2 attachment to ACE-2 receptors likely impairs ANG II hydrolysis, contributing to its unchecked accumulation. In addition, ADAM17 shedding of ACE-2 from the cell plasma membrane generates inactive, soluble ACE-2 (sACE-2) incapable of physiological functions (Patel et al., 2014). This engenders a vicious circle as excess ANG II activates ADAM17 that in turn upregulates ANG II by inhibiting its degradation. This may contribute to COVID-19 critical illness as overactive ADAM17 can lower the ACE-2 function by generating inactive sACE-2. Indeed, ACE-2 downregulation was associated with unfavorable COVID-19 prognosis, suggesting that sACE-2 along with the attached SARS-CoV-2 virus likely disseminates the infection throughout the body (Sfera et al., 2020; Verdecchia et al., 2020).

Dysfunctional renin-angiotensin system (RAS) was previously associated with accelerated aging as excess ANG II induces telomere attrition as demonstrated in both severe COVID-19 illness and children with Hutchinson-Gilford progeria, a syndrome of accelerated aging (Regenass et al., 1994; Herbert et al., 2008; Gerhard-Herman et al., 2012; Amraei and Rahimi, 2020; Benetos et al., 2020; Bidault et al., 2020; Libby and Lüscher, 2020). Moreover, ANG II-shortened telomeres and cellular senescence were documented in ME/CFS and cardiovascular disease, linking dysfunctional RAS to premature aging (Lieberman and Bell, 1993; Minamino and Komuro, 2002; Vasan et al., 2008; Fyhrquist et al., 2013; Squassina et al., 2019). Conversely, the telomere-repairing enzyme, telomerase, was reported not only to reverse ANG II-induced telomeres damage but also to ameliorate the symptoms of ME/CFS, further connecting this disorder to premature cellular senescence (Imanishi et al., 2005; Findeisen et al., 2011; Ho et al., 2012; Ait-Aissa et al., 2018). In addition, losartan, an ANG II receptor blocker (ARB), was demonstrated to restore the integrity of intestinal barrier as well as improve many symptoms of ME/CFS, further supporting the link between dysfunctional RAS and aging (Feng et al., 2011; Kumar et al., 2015; Nozu et al., 2020). Moreover, endothelial senescence and Alzheimer's disease (AD) were associated with ANG II-activated ADAM17, connecting both proteins to microvascular aging and disrupted biological barriers (Shatanawi et al., 2011; Morancho et al., 2015; Qian et al., 2016; Dou et al., 2017; Li et al., 2019; Shalaby et al., 2020). Indeed, impaired intestinal barrier with subsequent endotoxemia were demonstrated in both AD and Parkinson's diseases (PD) patients, suggesting that LPS could reach the CNS and initiate neurodegeneration (Hoban et al., 2013; Sun et al., 2016; Zhao et al., 2017; Shigemoto-Mogami et al., 2018). Along these lines, earlier studies demonstrated that LPS could access other organs, including the lung (via ANGII/ADAM17-increased vascular permeability) and trigger gram-negative pneumonias, connecting dysfunctional gut barrier to organ-specific pathology (Dreymueller et al., 2012; Morancho et al., 2015).

**Figure 2:**
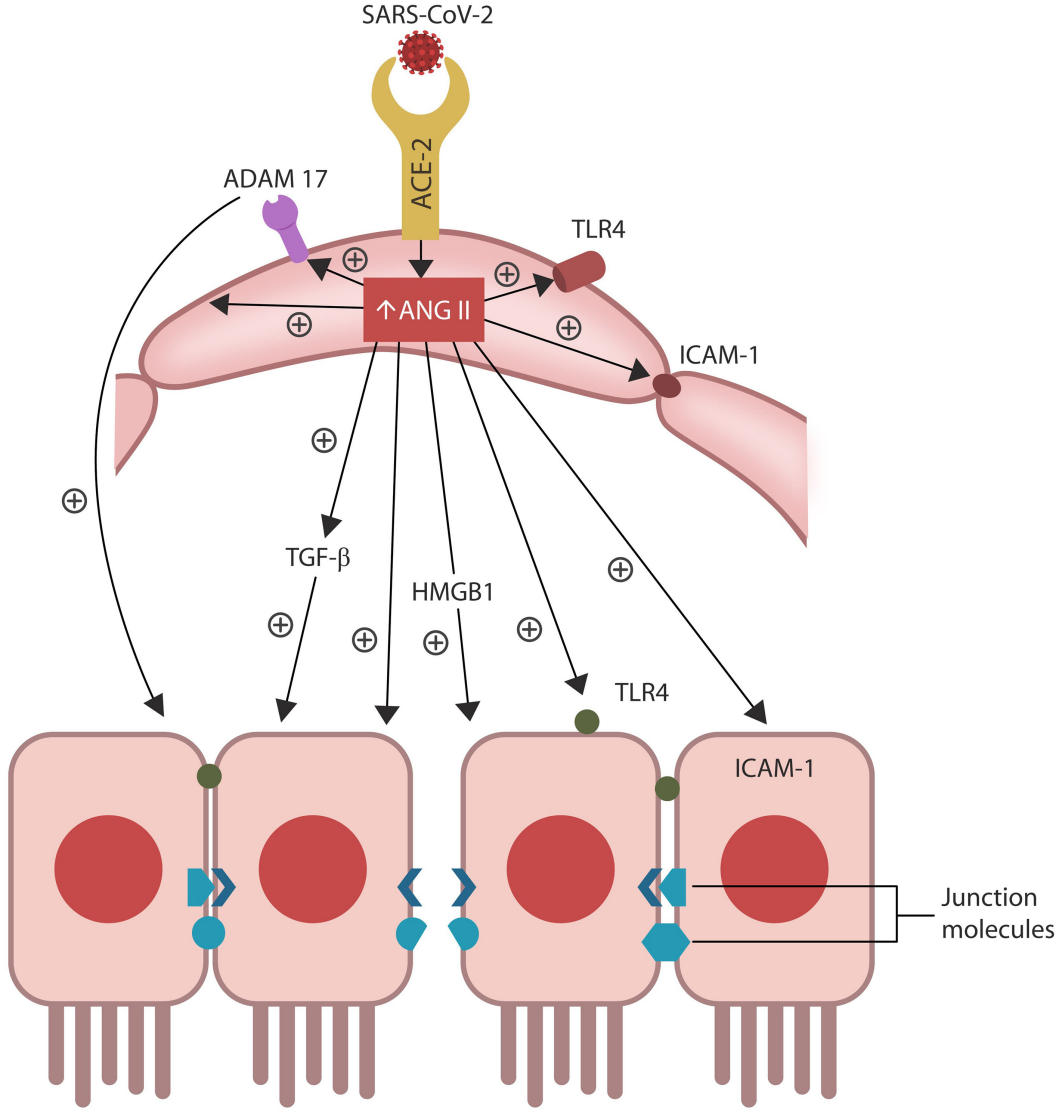
ANG II accumulation in ECs alters several senescence-associated pathways, including ADAM17-downregulation of ACE-2 in ECs and IECs. ANG II-upregulated pathways include TGF-β, HMGB1, TLR4, ICAM-1, and SASP (not shown). Together these molecules induce cellular senescence, increasing intestinal and BBB permeability likely contributing to ME/CFS.

Aside from activating ADAM17, ANG II also upregulates intracellular TGF-β, HMBG1, TLR4, and ICAM-1, promoting inflammation, fibrosis and oxidative stress that in turn, disrupt the biological barriers and immunity (Ribadeneira et al., 1996; Kunieda et al., 2006; Crowley and Rudemiller, 2017; Cooper et al., 2018; Figure 2). ANG II also inhibits the muscle tissue repair, generating myopathy and atrophy as well as endothelial and immune damage, further implicating dysfunctional RAS in ME/CFS symptoms (Ferrario et al., 2005; Turowski et al., 2005; Cabello-Verrugio et al., 2012; Morris et al., 2014; Wyller et al., 2016; Cooper et al., 2018; Tirone et al., 2018; Monteil et al., 2020; Figure 1). Indeed, defective endothelia were directly correlated with the severity of ME/CFS symptoms, emphasizing the role of defective vascular barrier in the pathogenesis of this disease (Yamazaki et al., 2016; Obrenovich, 2018; Scherbakov et al., 2020).

Senescent cells permanently exit the cell cycle but remain metabolically active, releasing pro-inflammatory cytokines that comprise a specific secretome, the senescence-associated secretory phenotype (SASP). ANG II upregulates SASP, while ADAM17 sheds these molecules from the cell plasma membranes, facilitating their dissemination throughout the body (Effenberger et al., 2014; Rajeevan et al., 2018; Aviv, 2020; Simões et al., 2020; Song et al., 2020; Takeshita et al., 2020).

Preclinical studies have reported that aside from disrupting ECs and the vascular barrier, ANG II can trigger IECs apoptosis, increasing also the permeability of the epithelial barrier, a condition often referred to as dysbiosis or “leaky gut” (Koga et al., 2008; Shi et al., 2016; Tanaka and Itoh, 2019; Figure 2). Indeed, a bidirectional relationship was found between RAS dysfunction and intestinal dysbiosis, suggesting that ARBs may be therapeutic by optimizing the barrier integrity (Lu et al., 2018; Viana et al., 2020).

### ANG II Independent Changes

The SARS-CoV-2/host interactome has revealed senescence-inducing mechanisms that can be activated by the direct cross talk between various viral antigens and host proteins. For example, mitochondrial damage can be inflicted indirectly by ANG II or by the direct cross talk of SARS-CoV-2 antigens ORF9C, NSP4, and NSP8 with mitochondrial proteins (Gordon et al., 2020; Table 1, Figure 1). In addition, aside from ANG II, telomere attrition can be triggered by the SARS-CoV-2 protein NSP5 interaction with human histone deacetylases 2 (HDAC2) or glutathione peroxidase (GPX) (Takakura et al., 2001; Månsson et al., 2019; Table 1, Figure 1).

Moreover, the dialog of SARS-CoV-2 antigen NSP6 or ORF9C with the human mammalian target of rapamycin complex 1 (mTORC-1) disrupts both IECs and myocytes, suggesting a role in the pathogenesis of ME/CFS (Yoon, 2017; Kaur and Moreau, 2019; Gordon et al., 2020). Likewise, the cross talk between viral protein ORF8 and host interleukin 17 (IL-17) was shown to alter the claudin molecule, disrupting intestinal barrier (Lee J. S. et al., 2015; Matsumoto et al., 2017; Veldhoen, 2017; Gordon et al., 2020). Indeed, SARS-CoV-2 manipulation of IL-17 may contribute directly to the pathogenesis of ME/CFS as this cytokine is crucial for muscle contractility and its depletion characterizes Duchenne muscular dystrophy (De Pasquale et al., 2012; Wang et al., 2019). Furthermore, the interaction between the N antigen of SARS-CoV-2 and host signal transducer and activator of transcription (STAT) 1 and 2, can disrupt both the function of skeletal muscle and anti-viral defenses, indicating that the virus exploits several pathways associated with weakness, fatigue and the susceptibility to infections (Moresi et al., 2019; Mu et al., 2020; Table 1).

## ANG II and Defective Efferocytosis

Novel studies have shown that aside from inducing cellular senescence, COVID-19 disrupts the clearance or efferocytosis of aging or dying cells, leading to their unchecked accumulation. Excessive buildup of senescent cells has been associated with exhaustion, fatigue, sarcopenia, frailty, immune dysfunction and cognitive impairment, symptoms that also characterize ME/CFS (Jeyapalan and Sedivy, 2008; LeBrasseur et al., 2015; Lee, 2019; Nelke et al., 2019; Martínez-Cué and Rueda, 2020). For example, chemotherapy-receiving breast cancer patients with elevated levels of p16INK4a senescent marker were found to experience most fatigue (Sanoff et al., 2014; Rajeevan et al., 2018). On the other hand, enhanced clearance of senescent cells was associated with improved physical activity and reduced fatigue in animal models (Demaria et al., 2017). For this reason, various senotherapeutic strategies targeting frailty and exhaustion in individuals on cancer therapy are currently in clinical trials, suggesting that these agents may also benefit ME/CFS patients (Lewis-McDougall et al., 2019; Short et al., 2019).

Under normal circumstances, billions of cells in the human body undergo apoptosis each day and are promptly removed by professional and non-professional phagocytes, including macrophages, NKCs and ECs (facultative phagocytes) (Vann and Proctor, 1990; Kirsch et al., 2007; Qingxian et al., 2010; Seeberg et al., 2019; Zhou et al., 2019). Macrophages, the primary efferocytosis executors, also participate in endothelial repair, linking the elimination of senescent cells to the pathology of biological barriers (Kearns et al., 2012; Zhu et al., 2016). On the other hand, ANG II signaling via angiotensin II type 1 receptors (AT-1Rs) disrupts both efferocytosis and ECs function as this peptide shifts macrophages from the phagocytic (M2) to proinflammatory (M1) phenotype, a pattern consistent with autoimmune inflammation (Belline et al., 2004; Yamamoto et al., 2011). ANG II disrupts the phagocytic function of macrophages and efferocytosis in addition to inducing ECs senescence and barrier dysfunction, defects encountered in both ME/CFS and COVID-19 critical illness (Zhang et al., 2019; Schulte-Schrepping et al., 2020). Conversely, the macrophage-activating factor was shown to restore normal efferocytosis as well as ameliorate many ME/CFS symptoms, suggesting a therapeutic potential (Inui et al., 2015). Moreover, both hyperinflammation and endotoxin tolerance, were demonstrated in ME/CFS and severe COVID-19, linking aberrant immune responses to the accumulation of senescent cell (Monneret and Venet, 2012; Fenwick et al., 2019; Zheng et al., 2020). Indeed, dysfunctional macrophages can generate a sepsis-like immune pattern marked by an initial hyperinflammation followed by immunosuppression, endotoxin tolerance and exhausted lymphocytes (Pena et al., 2011; Elder and Emmerson, 2020). As aberrant immune responses were associated with both ME/CFS and COVID-19 critical illness, dysfunctional efferocytosis is a likely contributor to both disorders (Manestar-Blazić and Volf, 2009; Morris and Maes, 2013; Fukushima et al., 2018; Rajeevan et al., 2018; Silva et al., 2019; Zhou T. et al., 2020; Kruglikov and Scherer, 2021).

### Efferocytosis: The Molecular Mechanisms

Efferocytosis is initiated by phagocytes responding to the “eat me” signals expressed on the plasma membrane of senescent or dying cells, marking them as “ready” for clearance (Barth et al., 2017; Karaji and Sattentau, 2017; Kale et al., 2020). Defective elimination of senescent ECs and IECs may increase the permeability of intestinal barrier, promoting dysbiosis and microbial translocation.

In general, efferocytosis occurs without immunogenicity as phagocytes engulf target cells without “spillage” of intracellular material into the extracellular milieu. When cytosolic content “escapes” cell confinement, it acts as a damage-associated molecular pattern (DAMP) that activates immunity, engendering inflammation (Abdolmaleki et al., 2018; Kawano and Nagata, 2018). For example, extracellular HMGB1 (a molecule upregulated by ANG II) is a potent DAMP associated with COVID-19 cytokine storm and several autoimmune disorders (Friggeri et al., 2010; Banerjee et al., 2011; Harris et al., 2012; Magna and Pisetsky, 2014; Tsung et al., 2014; Chen R. et al., 2020; Mangalmurti and Hunter, 2020; Figure 1). Interestingly, excessive HMBG1 was linked to unexplained fatigue, chronic pain, exhaustion, and muscle dysfunction, suggesting a role in the pathogenesis of ME/CFS (Morris and Maes, 2013; Zong et al., 2013; Wan et al., 2016; Nguyen et al., 2017; Figure 2).

Efferocytosis molecular sensors, including MerTK, recognize the externalized phosphatidylserine (PS), a major “eat me” signal, expressed by senescent or dying cell, earmarking them for clearance. MerTK is shed and inactivated by ADAM17, disrupting efferocytosis and triggering senescent cells-mediated pathology (Thorp et al., 2011; Dransfield et al., 2015; de Couto et al., 2019; Palau et al., 2020; Sfera et al., 2020; Figure 2). Indeed, overactive ADAM17 with excessive shedding of MerTK and ACE-2 could comprise a significant pathogenetic mechanism of COVID-19 and ME/CFS (Casciola-Rosen et al., 2020; Chaudhary, 2020; Megremis et al., 2020; Miesbach, 2020; Pagliaro and Penna, 2020). Along these lines, studies in athletes found a direct correlation between ACE-2 downregulation and poor muscle performance, further linking ADAM17 to the pathogenesis of ME/CFS (Motta-Santos et al., 2016). Interestingly, PS externalization was shown to promote ADAM17 activation and ACE-2 shedding, suggesting that the SARS-CoV-2 virus likely exploits this mechanism (Sommer et al., 2016).

### The SARS-CoV-2 Virus and Apoptotic Mimicry

It has been established that some viruses exploit PS signaling to directly invade the host phagocytes, a process known as apoptotic mimicry (Mercer and Helenius, 2010; Morizono and Chen, 2014; Segawa and Nagata, 2015). Emerging evidence indicates that the SARS-CoV-2 virus may disrupt efferocytosis by accessing host phagocytic cells by this route. Indeed, anti-PS antibodies, PS-containing extracellular vesicles (EVs) and platelets with externalized PS were demonstrated in COVID-19 critical illness (Urciuoli and Peruzzi, 2020; Zaid et al., 2020; Zhou Y. et al., 2020; Lind, 2021). Moreover, since ECs (non-professional phagocytes) express PS receptors, the SARS-CoV-2 virus may usurp these proteins to directly invade host endothelia (Setty and Betal, 2008). In addition, neutrophils with externalized PS were reported in ME/CFS patients, suggesting that viruses capable of apoptotic mimicry may contribute to this disorder (Kennedy et al., 2004). Furthermore, dysfunctional NKCs (professional phagocytes) were demonstrated in ME/CFS patients, further connecting this illness to apoptotic mimicry-impaired efferocytosis (Maher et al., 2005; Eaton-Fitch et al., 2019).

The SARS-CoV-2/host interactome found that the crosstalk between viral protein E (envelope) and human BRD-2 and BRD-4 triggers macrophage senescence and efferocytosis disruption (Gordon et al., 2020; Wang H. et al., 2020; Table 1). Interestingly, the SARS-CoV-2 spike (S) protein interacts with microbial LPS, inducing macrophage senescence, indicating that the virus may utilize several parallel mechanisms for disabling the efferocytosis (Petruk et al., 2020). Since BDR-4 is an established driver of angiogenesis and microvascular repair, viral exploitation of this protein likely alters the biological barriers, enabling microbial translocation and the ME/CFS pathogenesis (Maes and Leunis, 2008; Maes et al., 2014; Zhou Z. et al., 2020). Furthermore, BRD-4 depletion was associated with generalized muscle weakness, suggesting that post-exertional malaise, a well-established ME/CFS marker, may be engendered through this mechanism (Segatto et al., 2017).

Taken together, this data shows that the SARS-CoV-2 virus likely utilizes apoptotic mimicry to disrupt efferocytosis, leading to the accumulation of senescent cells that in turn triggers hyperinflammation which exhausts the immune system, facilitating viral infection. Impaired efferocytosis in the intestinal barrier and failure to eliminate senescent ECs and IECs may increase the gut permeability, allowing microbial/LPS translocation.

## Cellular Senescence and the Gut

Intestinal barrier separates the gut lumen from the rest of the body, preventing migration of microbes or molecules outside the GI tract, while at the same time, ensuring adequate nutrient absorption. A single layer of IECs covered by abundant mucus comprise the epithelial portion of the gut barrier, while ECs constitute the vascular component (Thomas, 2016). Aside from the barrier function, IECs mediate the interaction between gut microorganisms and resident immune cells, balancing the immunological acceptance of intestinal microbes with pathogen rejection (Mizrahi and Ilan, 2009; Edelblum et al., 2017; Poggi et al., 2019). At the molecular level, IECs are kept together by junction molecules, including the claudins, that control barrier permeability by regulating the size of intercellular spaces (Garcia-Hernandez et al., 2017; Garcia et al., 2018; Figure 2). Interestingly, preclinical studies have reported that ANG II, acting via AT-1R, increases the permeability of intestinal barrier by altering the expression of claudin-7 (Shi et al., 2016; Takashina et al., 2020). In addition, IL-17, a cytokine directly usurped by the SARS-CoV-2 viral protein ORF8, influences the gut permeability via claudins (Lee J. S. et al., 2015; Andrews et al., 2018; Table 1). Moreover, the SARS-CoV-2 antigens NSP4, NSP8, and ORF9C interact with IECs mitochondria increasing the permeability of intestinal barrier by an alternative mechanism (Lee J. H. et al., 2015; JanssenDuijghuijsen et al., 2017; Figure 1, Table 1). Moreover, ICAM-1, an ANG II-controlled protein, is essential for maintaining intestinal and endothelial integrity, suggesting that the SARS-CoV-2 virus can also alter the gut barrier by manipulating RAS (Sumagin et al., 2014; Sarelius and Glading, 2015; Figure 2).

### The Permeability-Immunity Axis

In the GI tract, intestinal permeability is tightly intertwined with local immunity as IECs, ECs and gut resident immune cells regulate the barrier function, nutrient absorption and gut microbial composition. For example, intestinal ACE-2 receptors also function as neutral amino acid transporters (B0AT1 or SLC6A19), therefore a dysfunctional RAS can trigger barrier disruption, dysbiosis and amino acid malabsorption (Cheng et al., 2017; Jando et al., 2017; Viana et al., 2020). For this reason, it is not surprising that COVID-19 and ME/CFS have been associated with both aberrant immune responses and dysfunctional intestinal permeability (Gaab et al., 2005; Maes and Leunis, 2008; Maes et al., 2014; Morris et al., 2019). Conversely, normalization of gut barrier was found to ameliorate immunity in COVID-19 and the symptoms of ME/CFS patients (Maes and Leunis, 2008; Maes et al., 2014; Du Preez et al., 2018; Mandarano et al., 2018; Cardinale et al., 2020). Along these lines, the permeability-immunity connection may explain the higher prevalence of inflammatory bowel disease (IBD) in ME/CFS patients as both conditions are marked by dysfunctional gut barrier and immune responses (Newton et al., 2012; Gravina et al., 2018; Mandarano et al., 2018; Tsai et al., 2019; Scherbakov et al., 2020). With the same token, active IBD was associated with unfavorable COVID-19 outcomes, suggesting that the SARS-CoV-2 attachment to ACE-2 (abundantly expressed on IECs and resident Tregs) disrupts both the intestinal barrier and the local immunity (Bezzio et al., 2020; Lin et al., 2020). In addition, the premature senescence of intestinal endothelia and increased microbial translocation may contribute to body-wide immune changes demonstrated in ME/CFS and COVID-19 critical illness (Poujol et al., 2015; Huth et al., 2016; Kasper et al., 2016; Mandarano et al., 2018; Galván-Peña et al., 2020; Li et al., 2020; van Eeden et al., 2020; Zheng et al., 2020).

In the GI tract, commensals are immunologically protected by the resident Tregs that mediate luminal immunosuppression. However, outside the gut, microbes are vigorously attacked by the host immune system that often generates hyperinflammatory responses that may exhaust the lymphocytes, generating endotoxin tolerance (Ramos et al., 2016; Sotzny et al., 2018; Sepúlveda et al., 2019; Fine et al., 2020).

At the molecular level, Tregs-induced immunosuppression is engendered by the NRP-1/IL-10 signaling that requires the presence of vascular endothelial growth factor (VEGF) (Ohm et al., 2003; Wang et al., 2015; Chuckran et al., 2020). The SARS-CoV-2 attachment to NRP-1 lowers VEGF, leading to excessive immune tolerance that helps the virus avert detection (Yin et al., 2020; Mayi et al., 2021). In addition, as VEGF also regulates angiogenesis, endothelial senescence and efferocytosis, the disruption of NRP-1/IL-10/VEGF axis likely triggers the ME/CFS and COVID-19 pathogenesis (Watanabe et al., 1997; Hasan et al., 2011; Kearns et al., 2012). Moreover, SARS-CoV-2/NRP-1 attachment and dysfunctional VEGF in IECs, myocytes, ECs and Tregs was associated with disabling fatigue, further connecting these proteins to ME/CFS (Yadav et al., 2012; Wang et al., 2015; Yamaji et al., 2015; Issitt et al., 2019; Cantuti-Castelvetri et al., 2020; Davies et al., 2020; Moutal et al., 2021; Table 1). Interestingly, earlier studies have reported VEGF downregulation in ME/CFS patients, linking this growth factor to disabling fatigue (Landi et al., 2016; Dai et al., 2017; Petruk et al., 2020). Moreover, as the S antigen of SARS-CoV-2 virus binds microbial LPS, and LPS-activated Tregs inhibit immune responses, the ME/CFS-associated endotoxin tolerance may be triggered via this mechanism (Lewkowicz et al., 2006; Morris et al., 2019; Table 2). Furthermore, the SARS-CoV-2 virus can also generate endotoxin tolerance in other manner, including ANG II-upregulation of intracellular HMGB1 and TGF-β, NSP6/ORF9C interaction with host mTORC-1, and antigen E cross talk with BRD-2 and BRD-4 (Aneja et al., 2008; Yang et al., 2015; Sun et al., 2018; Copsel et al., 2019; Figure 1, Table 2).

**Table 2:** The SARS-CoV-2 immunological tolerance-inducing mechanisms.

**Mechanism**	**Endotoxin tolerance**
ANG II	HMGB-1 TGF- β
NSP6 ORF9C	mTORC-1
E	BRD-2 BRD-4
LPS VEGF	Tregs activation

*It is noteworthy that Tregs activation lowers immune responses, likely generating endotoxin tolerance*.

### Autoantibodies in ME/CFS

Another manifestation of the ME/CFS-associated immune dysregulation is the presence of autoantibodies directed against cholinergic and β2 adrenergic receptors (β2AdRs) documented by numerous studies (Loebel et al., 2016; Wirth and Scheibenbogen, 2020). Autoantibodies are believed to reflect the presence of altered “self” proteins that are unrecognized and therefore attacked by the immune system.

An alternative explanation could fathom autoantibodies in ME/CFS patients as being directed at microbial molecules translocated from the host GI tract. Indeed, many gut microbes express muscarinic and adrenergic receptors, suggesting that autoantibodies may target these molecules (Furukawa and Haga, 2000; Karavolos et al., 2013; Moreira et al., 2016). Along these lines, translocated microorganisms that express β2AdRs, may elicit antibodies cross-reacting with host's own adrenergic receptors. For example, antibodies against β1 and β2AdRs of translocated Escherichia coli may cross react with their human receptors (Freissmuth et al., 1991). Interestingly, recent studies have demonstrated that many ME/CFS patients present with significantly higher norepinephrine plasma levels, indicating that anti-β2AdR antibodies may aim at downregulating adrenergic transmission and restore homeostasis (Wyller et al., 2016).

Under normal circumstances, the anti-β2AdR antibodies activate β2AdR receptors, however (probably due to elevated norepinephrine) this response is attenuated in ME/CFS patients, suggesting once more the compensatory role of autoantibodies (Hartwig et al., 2020). Other pathogens, including the reovirus type 3, demonstrated molecular mimicry with human β2AdR, promoting adaptive autoantibodies that eliminate the virus-infected cells by targeting these receptors (Co et al., 1985). Moreover, a recent study demonstrated that the microbial metabolite phenylacetylglutamine (PAGIn) excessively upregulates human cardiac adrenergic signaling, promoting heart disease, a condition that autoantibodies could preempt (Nemet et al., 2020). With the same token, viruses that populate the human GI tract were shown to modify various host molecules, transforming them into immune targets (Campbell, 2014; Mukhopadhya et al., 2019). For example, the hemagglutinin antigen (HA) of H1N1 influenza virus can alter host hypocretin molecule, turning it into an antigen significant for the pathogenesis of autoimmune narcolepsy (Luo et al., 2018). On the other hand, increased tryptophan absorption due to dysfunctional intestinal barrier and overactivation of brain kynurenine pathway (KP) was demonstrated in ME/CFS patients, further linking the dysfunctional GI tract to this disease (Georgiades et al., 2003; Yamashita, 2020). Interestingly, ARBs were demonstrated to inhibit KP, probably by normalizing intestinal permeability, limiting tryptophan absorption (Blankfield, 2011; Zakrocka et al., 2017).

## Potential Interventions for Barrier Dysfunction

This section focuses on three types of interventions in line with the hypothesis presented here: restoration of adequate intestinal permeability, LPS lowering or removal and efferocytosis optimization. Most agents operate by more than one mechanism of action, indicating that superior efficacy may be achieved by combining therapeutic modalities. Indeed, some ME/CFS drugs currently in use directly or indirectly restore the function of biological barriers, ameliorate efferocytosis and lower LPS. For example, rituximab, a monoclonal antibody often utilized in ME/CFS, improves the phagocytic function of macrophages and efferocytosis, in addition to its established actions on antibodies and B lymphocytes (Toubi et al., 2007; Djaldetti et al., 2019). Another example is escitalopram, a frequently prescribed drug to ME/CFS patients with depressed mood, that aside from its antidepressant action, also promotes endothelial restoration, optimizing the permeability of biological barriers (Lopez-Vilchez et al., 2016). Other ME/CFS therapies, including the combination of coenzyme Q10 and nicotinamide adenine dinucleotide (NAD), improve endothelial function, demonstrating an alternative mechanism of action (Gao et al., 2012; Castro-Marrero et al., 2016; Mateuszuk et al., 2020). Conversely, drugs that restore endothelial integrity, including beta blockers, are often beneficial to ME/CFS patients, emphasizing the role of dysfunctional biological barriers in the pathogenesis of this illness (Su, 2015; Wyller et al., 2016). Mildronate, an anti-ischemic drug, often helpful to ME/CFS patients, also restores endothelial integrity by upregulating nitric oxide (Sjakste et al., 2005; Comhaire and Deslypere, 2020). Yet other endothelium-protecting drugs, such as ARBs, may offer relief to ME/CFS patients as they improve muscle strength, exercise capacity, and cognition (Nade et al., 2015; Coelho et al., 2016). In addition, ARBs optimize intestinal permeability and macrophage-mediated efferocytosis, indicating more than one action mechanism (Villapol and Saavedra, 2015; Shi et al., 2016). Moreover, a cross-reaction was demonstrated between AT-1Rs and β2AdRs, as they are inhibited by a single antagonist, suggesting that ARBs and β-blockers may be equally effective in ME/CFS (Blumenfeld et al., 1999; Barki-Harrington et al., 2003).

Considering the hypothesis presented here, several drugs not currently utilized in ME/CFS may emerge as potential therapies. These include short chain fatty acids (SCFAs), milk fat globule membranes (MFGM), β-glucan, VEGF-agonists, fecal microbial transplantation and senolytic agents, including Navitoclax and fistein.

Microbial products, such as SCFAs are derived from the fermentation of dietary fiber in the GI tract. They may be beneficial to ME/CFS patients as they promote intestinal barrier restoration, correct dysbiosis and local immunity (Chen et al., 2017; Newberry et al., 2018; Yang et al., 2020).

Fecal microbial transplantation (FMT), has been suggested as a treatment modality for ME/CFS after being promoted by a few uncontrolled studies, that found long lasting improvement (Castro-Marrero et al., 2016). However, at present, the application protocols, optimal donors and the long-term risks of FMT are not entirely clear (Evrensel and Ceylan, 2016).

Milk fat globule membranes (MFGM) and β-glucan were found to decrease both microbial translocation and fatigue in murine models (Vetvicka and Vetvickova, 2015; Vetvicka et al., 2019; Yu et al., 2020). Indeed, aside from restoring the intestinal barrier, β-glucan also reverses endotoxin tolerance, correcting the aberrant immune responses that characterize ME/CFS (Wu et al., 2018).

Upon crossing from the GI tract into the systemic circulation, LPS may access the brain via disrupted BBB or areas of high physiological permeability, such as the circumventricular organs. Inside the brain, endotoxin binds to microglial TLR4, activating these cells, probably contributing to cognitive impairment, or “brain fog” experienced by many ME/CFS patients (Qin et al., 2007; Theoharides et al., 2015; Tsukamoto et al., 2018). β-glucan inhibits TLR4 endocytosis, preventing LPS activation of microglia and the subsequent cognitive impairment (Novakovic et al., 2016). Interestingly, LPS-binding protein (LBP), an acute phase reactant, can remove LPS by attaching to it tightly, neutralizing its actions, suggesting a therapeutic potential in ME/CFS (Mathison et al., 1992; Goldblum et al., 1994; Giloteaux et al., 2016). Moreover, β-glucan was demonstrated to augment macrophage-mediated efferocytosis in animal model, indicating that by decreasing the accumulation of senescent cells it could help the ME/CFS patients (Fatima et al., 2017). Others have suggested that metformin protects the gut barrier by lowering LPS-inflicted damage, indicating a mechanism of action in line with the hypothesis presented here (Brown et al., 2018; Wu et al., 2018). Aside from safeguarding the GI tract, metformin also augments microglia-mediated efferocytosis, protecting the CNS against senescent cell pileup (Tabuzek et al., 2010).

A recent open-label, pilot study in human subjects utilized senotherapeutics in patients with pulmonary fibrosis, demonstrating good tolerability and indicating potential therapeutic benefits in ME/CFS (Justice et al., 2019). Indeed, several senolytic drugs were recently tested, including dasatinib (FDA-approved for chronic myeloid leukemia), hyperoside, quercetin, fistein and the BCL-2 inhibitor, Navitoclax (Kirkland et al., 2017; Mohamad Anuar et al., 2020). As ECs are dependent on BCL-2, Navitoclax should be tested for ME/CFS (Zhu et al., 2016; Wissler Gerdes et al., 2020). Fistein, currently in phase 2 clinical trials for the frail elderly syndrome, has demonstrated good tolerability, indicating potential beneficial effects in ME/CFS (AFFIRM-LITE trial NCT03675724).

## Conclusions

The data presented above supports the hypothesis that ME/CFS pathology likely commences with a pathogen-induced intestinal barrier disruption and subsequent microbial translocation. These in turn trigger aberrant immune responses in various host tissues, ranging from autoimmune inflammation (cytokine storm) to excessive tolerance, likely engendering the COVID-19 and ME/CFS pathologies.

Restoration of adequate intestinal permeability, LPS lowering and efferocytosis optimization are the suggested interventions in line with the hypothesis presented here.

## Limitations

This is not a systematic review hence additional publications might be identified as relevant to the subject. Secondly, while this hypothesis has a clear biological foundation, it would still require to be validated at a minimum through retrospective analysis of clinical data where possible. Thirdly, as the SARS-CoV-2 interactome has demonstrated, systemic infections present a complicated landscape or pathways that may act synergistically or in parallel. As such this hypothesis can be viewed as a starting point toward an increased understanding of the relationship between viral infections, chronic inflammation (including that of the gut) and ME/CFS.

## Future Perspectives

ME/CFS is a serious illness with unclear etiology and non-specific treatments. In addition, as it is often dismissed by both the public and healthcare workers, patients with this condition are frequently stigmatized and may avoid seeking help. Decreasing stigmatization by educating the public and clinicians on the biological aspects of this condition is therefore very important. In this regard, the molecular hypothesis presented here may contribute to this goal.

Future research will likely probe deeper into the interface between gut microbes and the local immune system, elucidating not only the pathogenesis of ME/CFS, but also of other illnesses associated with dysfunctional immune tolerance or activation, including autoimmune, neuropsychiatric and degenerative disorders.

Studying the virus/host interactome and associated pathology will contribute to a better understanding of the largest immune compartment in the body, the GI tract, and its role in the immunological acceptance of gut microbes. This will likely contribute to the development of antigen-specific immunosuppressant therapies, such human Tregs. Indeed, allogeneic T cells are currently in clinical trials for COVID-19 hyperinflammatory syndrome (NCT04482699). Unlike non-specific immunosuppressive agents that impact many tissues and organs, generating adverse effects, Tregs offers specificity and precision that could benefit not only the patents with ME/CFS, but also those with allergies, transplants and infectious diseases.

Furthermore, elucidation of the molecular underpinnings of microbial translocation will undoubtedly lead to more specific treatments for restoring the adequate permeability of the GI tract, that would benefit patients with inflammatory bowel disease (IBS). As gut microbes alter both the intestinal and blood-brain barrier, manipulation of microorganismal translocation will likely contribute to the development of treatments for some CNS diseases, including the neurodegenerative disorders, such as Alzheimer's and Parkinson's disease (Osorio et al., 2019).

## Data Availability Statement

The original contributions presented in the study are included in the article/supplementary material, further inquiries can be directed to the corresponding author/s.

## Author Contributions

All authors listed have made a substantial, direct and intellectual contribution to the work, and approved it for publication.

## Conflict of Interest

The authors declare that the research was conducted in the absence of any commercial or financial relationships that could be construed as a potential conflict of interest.
